# Behavior Change Techniques in Popular Mobile Apps for Smoking Cessation in France: Content Analysis

**DOI:** 10.2196/26082

**Published:** 2021-05-13

**Authors:** Luz Adriana Bustamante, Cédric Gill Ménard, Sabrina Julien, Lucia Romo

**Affiliations:** 1 Laboratoire EA 4430-Clinique Psychanalyse Developpement Department of Psychology University of Paris Nanterre Nanterre, Ile-de-France France; 2 C2S - Cognition Santé Société (EA 6291) Department of Psychology Université de Reims Champagne - Ardenne Reims France

**Keywords:** smartphone app, smoking cessation, mHealth, app quality, user engagement, behavior change technique taxonomy

## Abstract

**Background:**

The mobile app market differs from country to country, and to date, no previous review of the content quality of smoking cessation apps has been conducted in France.

**Objective:**

This study aimed to examine the general quality of the most popular smoking cessation apps in France and also determine the degree to which apps adhere to established behavioral and cognitive techniques (BCTs) proven effective in clinical practice.

**Methods:**

A systematic research of smoking cessation apps was conducted in both the Google Play Store and Apple Store in the French market. The general quality of popular apps was rated with the Mobile App Rating Scale (MARS), and the therapeutic quality was assessed with the ratio of adherence of the behavior change technique taxonomy for smoking cessation treatment.

**Results:**

A total of 14 mobile apps met all the inclusion criteria of the content analysis. The interrater reliability varied from “substantial” (0.79) to “almost perfect” (0.9) for the two measures. The mean MARS score was 3.5 out of 5 (median 3.6, IQR 0.6 [3.2-3.8]). The findings suggest that popular apps focus primarily on the functionality dimension of the MARS scale (4.2/5). The mean number of BCTs was 22, with a large difference between apps (minimum 4, maximum 38). At least half of the apps addressed motivation (8.8/14, 63%) and advised on using behavioral skills in order to quit smoking or stay a nonsmoker (8.7/14, 62%). However, only a handful of apps gathered important information (5.9/14, 42%) in order to deliver proper advice regarding the use of approved medication or the implementation of behavioral techniques (4.3/14, 31%). The mean MARS score was positively correlated with the price (*r*=0.70, *P*=.007) and the number of BCTs used (*r*=0.67, *P*=.01). User rating was not correlated with any quality scale (*P*=.67).

**Conclusions:**

The content quality of popular smoking cessation apps in France varied by app type and price. Most popular apps propose in general good quality content but lack implementation of evidence-based BCTs associated with effectiveness on smoking cessation treatment. Further research is needed to evaluate the improvement in the content quality of smoking cessation apps in France.

## Introduction

Despite a significant decrease in tobacco consumption in France from 30% in 2000 to 25.4% in 2018 and implementation of corresponding antismoking policies [1] and proven effective treatments [2], the prevalence of smokers aged 18 to 75 years is still a public health issue [1].

Among the new solutions proposed, mobile apps appear to be a promising form of support [3,4]. By adapting and transposing therapeutic principles already proven to be effective, apps may offer multiple benefits for patients, health care professionals, and the health care system itself [5-7]. One of the distinct mobile app benefits is ease of access to health care and therapeutic information [6]. In addition, an app-based digital health approach would help individuals foster a sense of responsibility and commitment to their own personal health through “nudges” like positive reinforcement via messaging, tracking of habits and regular feedback, and audiovisual support [5,7,8].

Although research on mobile apps is growing, it still lags behind innovation and business expansion [9]. The number of available smoking cessation apps is growing, as is their user base. For example, in 2009, there were 62 such apps available in the US market, and 3 years later, the number had quadrupled to 252 [10,11]. The number of apps has surely grown since then and far surpasses the number of peer-reviewed studies conducted in the same time period. As a Cochrane review notes, despite this proliferation of smoking cessation apps, there is insufficient evidence to conclude that they have a significant positive effect on long-term smoking cessation [12], even if studies conducted specifically on text message interventions have shown efficacy in increasing smoking cessation rates by 50% to 60% [12].

Beyond a relative dearth of studies, one factor that may explain the uncertainty surrounding the efficacy of these apps is that many do not integrate therapeutic approaches that have already been proven effective in other contexts. Indeed, all apps studied in the Cochrane review used different cognitive and behavioral theories as their point of departure [12,13]. The “active ingredients” (a term used to encapsulate the various strategies and practices of evidence-based behavioral and cognitive therapies) of strategies that have withstood clinical study and peer review and that are often recommended by public health authorities for clinical practice are not well integrated into the various apps available, whether in the American [11,14], Australian [15], or British [16] market.

Moreover, even if an app includes all the therapeutic guidelines, it might not be used; thus, its effectiveness would be limited. As Nielsen points out, 25% of the most downloaded apps are never used and 38% of purchased apps are immediately uninstalled after their first use [17]. The quality of user experience can also impact an app’s efficacy. Undoubtedly, “adherence to therapies is a primary determinant of treatment success” [18], and health apps are unlikely to be an exception. Therefore, it is essential to identify and assess the main factors underlying the quality of user experience, defined by O’Brien and Toms as “user engagement with technology” [19]. To date, several factors that interfere with the quality of user experience have been identified, and alternative forms of assessment have been proposed [20]. For example, the Mobile App Rating Scale (MARS) proposes the following four quality dimensions: engagement, functionality, esthetics, and information [20]. This scale has been used to assess the quality of various mobile health (mHealth) apps in diverse health fields from weight management [21] and drug interaction [22] to smoking cessation [15].

The mobile app market differs from country to country, and to date, no previous review of smoking cessation app content has been conducted in France. This means the findings of previous reviews on English-language apps may not be transposable to a French context. Besides, most studies do not take into account both the user experience and the therapeutic aspect to assess the quality of existing mobile smoking cessation apps. This review aimed to use the MARS to examine the general quality of the most popular iOS and Android smoking cessation apps and use the behavior change technique taxonomy to determine the adherence degree to established behavioral and cognitive techniques (BCTs) proven effective in clinical practice [23].

## Methods

### Study Design

The apps analyzed in this study were searched for and downloaded in France using both the iOS and Android app stores. The names of all apps and their descriptions were initially screened by the first author (LAB). Most of the apps were downloaded for a second screening, and only those that met all criteria were recorded on video as if the user had downloaded them for the first time. Based on these videos, two independent raters assessed the presence of BCTs and mobile app quality. The raters were all trained in health behavior change, and they are both behavioral and cognitive psychologists and researchers. Since subjects were not recruited, no ethics approval was required. The recorded videos were necessary to ensure that both raters assessed the same versions of the apps and to facilitate discussion.

### Sample

The study included both free and paid apps that support the French language and that intend to assist users with smoking cessation. The apps were identified on two occasions by the first author (LAB) (December 1, 2019, and April 20, 2020), using the app search keywords “smoking cessation,” “stop smoking,” and “quitting tobacco” (in French, “sevrage tabagique,” “arrêter de fumer,” and “arrêt tabac,” respectively). To be included in the full review, apps had to be designed to target smoking cessation only and support the French language. Excluded apps were those that were last updated before January 2019 and had ratings of less than 3/5 points in the app stores. Since each store provides different information on the number of downloads, we used different characteristics to select the most popular apps for a detailed content analysis. For android apps, where the number of downloads is available, an arbitrary threshold of 50,000 downloads was required to meet our popularity criteria, and since Apple Store does not share the number of app downloads, for iOS apps, an arbitrary threshold of 50 raters per app was set. Smokers wanting to quit with an app seem to favor apps with a strong “social proof,” meaning they care about the rating as well as the number of raters and number of downloads [24].

### Procedure: Coding of Apps

After the first author used and recorded each app, both raters assessed the apps’ content independently using both the MARS and behavior change technique taxonomy for smoking cessation. Prior to evaluation, all raters read each type of measure and had the opportunity to clarify and discuss the definitions in order to ensure clear differentiation between items.

#### MARS

The MARS is a multidimensional measure for classifying and assessing the quality of mobile apps. The MARS total score can be used to evaluate and compare the quality of an app with others, while the subscale “subjective quality” can be used to describe the strengths and weaknesses of a specific app. The total score is calculated by averaging the mean scores of the following five categories: user engagement, functionality, esthetics, information, and subjective quality. Each category is rated using a 5-point scale ranging from inadequate (1) to excellent (5) [20]. This scale has already been used for assessing the quality of smoking cessation apps in the Australian market, with a high interrater reliability (IRR) between raters (interclass correlation coefficient [ICC]=0.807) [15]. The scale is largely used and translated in different languages with a high intraclass correlation coefficient and good internal consistency [25,26].

#### Behavior Change Technique Taxonomy

The behavior change technique taxonomy for individual behavioral support for smoking cessation was used in this study [27]. A dichotomous score of “0” (absent) or “1” (present) was applied for each technique during the assessment of every app [21,27]. Each technique was classified within the following functions that are needed to ensure the efficacy of cognitive and behavioral therapy for smoking cessation: (1) focus on behavior (B), (2) addressing motivation (M), (3) maximizing self-regulatory skills (S), (4) promoting adjuvant activities (A), (5) general aspects of interaction (R), (6) information gathering (I), (7) general communication (C), and (8) delivery of the intervention (D) [27].

### Statistical Analysis

The statistical analysis was performed using IBM SPSS statistics version 26.0 (IBM Corp). Following the suggestion of “issues and best practices in content analysis” [28], we decided to calculate three measures of reliability for each scale. To assess the level of agreement between raters (IRR), we used the ICC and Krippendorff alpha for both scales, weighted kappa for the MARS, and prevalence and adjusted kappa for the behavior change technique taxonomy. The ICC was assessed in a two-way random model for an agreement level. The weighted kappa was assessed by pulling quadratic weights for each value. All reliability tests were performed per dimension of each scale and for all apps. Descriptive analysis was used to identify the presence of app characteristics (ie, mean price and frequency of BCTs), and one-way analysis of variance (ANOVA) and the post-hoc Tukey honestly significant difference (HSD) test were used to determine any significant difference observed between the scales. The mean score by dimension of each scale was used in the Spearman correlation test to examine the relationship among the price per month, user ratings, and both mobile app qualities (general and therapeutic).

## Results

### Systematic Search Results

**Figure 1:**
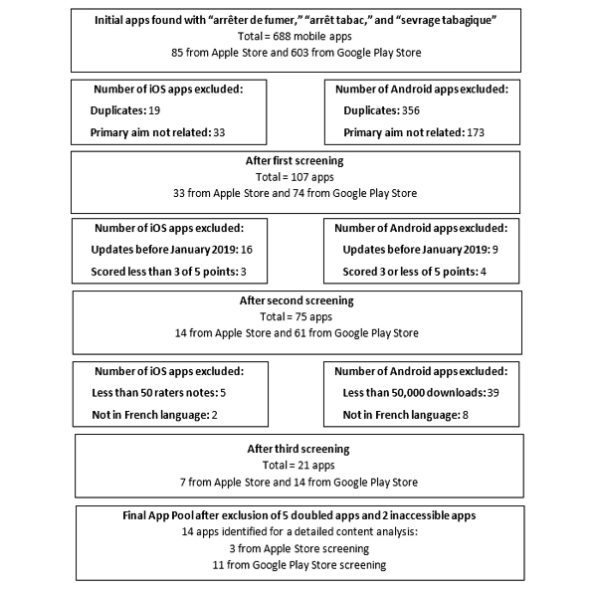
Flowchart of the results from the app search, preliminary inclusion and exclusion screening, and final app pool.

A total of 688 apps were initially identified from the French Google Play Store (n=603) and Apple Store (n=85). Figure 1 shows the results of the key stages of the mobile app review. After preliminary inclusion and exclusion criteria were applied, 74 Android and 33 iOS apps remained. Further screening based on evaluation of the app product page and the last update resulted in 61 Android and 14 iOS apps. Finally, further exclusion of apps upon download and during the coding procedure resulted in a total of 14 apps. Among these 14 apps, seven were accessible in both stores, six were accessible in the Google Play Store only, and one was accessible in the Apple Store only (Multimedia Appendix 1). 

### General App Characteristics

Table 1 shows that most of the included apps (12/14, 86%) were affiliated with a commercial company, while 14% (n=2) were affiliated with a university (n=1) and a government department (n=1). The mean user rating was very good (mean 4.5, range 3.7-4.8) in both stores without a particular difference. All apps were free to download and to use for a limited time or with limited features, but half of them had in-app purchases with different payment options that offered full access to all the contents of the app. The mean monthly price was €3.47 (US$ 4.19) in both stores, without a difference between both stores. Most apps included calculator (14/14, 100%), tracker (13/14, 93%), and information (13/14, 93%) features, as well as media connectors and reminders to use the app or to stay a nonsmoker. Very few apps (4/14, 29%) allowed users to protect their information with a password. All the apps needed internet access to download, and later, most of the features could be used offline.

**Table 1:** General information of the rated apps from the French Apple Store and Google Play Store (N=14).

Variable			Total (n=14)		iOS (Apple Store) (n=8)		Android (Google Play Store) (n=13)
**Affiliation, n (%)**							
	Commercial	12 (86%)		6 (75%)		11 (85%)	
	Unknown	0 (0%)		0 (0%)		0 (0%)	
	Government	1 (7%)		1 (13%)		1 (8%)	
	NGO^a^	0 (0%)		0 (0%)		0 (0%)	
	University	1 (7%)		1 (13%)		1 (8%)	
**Country of origin, n (%)**							
	England	1 (7%)		1 (13%)		1 (8%)	
	Spain	1 (7%)		1 (13%)		1 (8%)	
	France	4 (29%)		4 (50%)		1 (8%)	
	Switzerland	1 (7%)		1 (13%)		1 (8%)	
	Germany	4 (29%)		1 (13%)		4 (31%)	
	Ukraine	2 (14%)		0 (0%)		2 (15%)	
	United States	1 (7%)		0 (0%)		1 (8%)	
**Price**							
	Free, n (%)	6 (43%)		3 (38%)		6 (46%)	
	In-app payment, n (%)	8 (57%)		3 (38%)		7 (54%)	
	Price per month (€), mean (range; SD)	3.03 (0-9.99; 3.4)		3.68 (0-9.99; 3.5)		3.03 (0-9.99; 3.4)	
**Technical aspects, (%)**							
	Allows sharing	14 (100%)		8 (100%)		13 (100%)	
	Community	9 (64%)		7 (88%)		9 (69%)	
	Password protection	4 (29%)		3 (38%)		4 (31%)	
	Requires login	10 (71%)		6 (75%)		10 (77%)	
	Sends reminders	14 (100%)		8 (100%)		13 (100%)	
	Web access function	0 (0%)		0 (0%)		0 (0%)	
**Specific features, n (%)**							
	Calculator	14 (100%)		8 (100%)		13 (100%)	
	Rationing	2 (14%)		0 (0%)		1 (8%)	
	Tracker	13 (93%)		7 (88%)		12 (92%)	
	Information	13 (93%)		7 (88%)		13 (100%)	
	Game	6 (43%)		4 (50%)		6 (46%)	
	Lung health monitor	0 (0%)		0 (0%)		0 (0%)	
	Other	6 (43%)		5 (63%)		6 (46%)	
**Popularity**							
	User rating, mean (range)	4.5 (3.7-4.8; 0.32)		4.4 (4.1-4.7; 0.21)		4.4 (3.7-4.8; 0.32)	
	Number of ratings, mean (range)	—^b^		16,031 (74-83,000)		21,701 (221-86,713)	
**Store**							
	Apple only	1 (7%)		N/A^c^		N/A	
	Google Play only	6 (43%)		N/A		N/A	
	Both stores	8 (57%)		N/A		N/A	

^a^NGO: nongovernmental organization.

^b^Not possible to measure.

^c^N/A: not applicable.

### General Quality: MARS

The general quality was acceptable. The mean MARS score was 3.5 (median 3.6, IQR 0.6 [3.2-3.8]), with a maximum score of 4.3 and a minimum score of 2.4 (Table 2). Since only one app was not available in both the Google Play Store and Apple Store, no comparison between these distribution services was conducted. The IRR between the two raters was substantial. The ICC was 0.79 (95% CI 0.74-0.84), weighted kappa was 0.79 (95% CI 0.74-0.84), and Krippendorf alpha was .88 (95% CI .85-.91). Detailed results are presented in Multimedia Appendix 2.

**Table 2:** Quality of smoking cessation mobile apps in the French market according to the Mobile App Rating Scale (MARS).

Mobile App Rating Scale (MARS) category	Mean (SD) score	Score range (minimum-maximum)	IQR (Q1-Q3)
Engagement (fun, interest, interactivity...)	3.0 (0.8)	(1.8-4.5)	(2.4-3.4)
Functionality (app functioning, easy to learn...)	4.2 (0.8)	(1.9-4.9)	(4.0-4.8)
Esthetics (overall visual appeal, stylistic consistency...)	3.5 (0.6)	(2.3-4.5)	(3.2-4.0)
Information (text, feedback, measures...)	3.2 (0.6)	(2.3-4.4)	(2.8-3.6)
Subjective (recommendation, overall rating...)	2.1 (0.9)	(1.0-3.8)	(1.3-2.6)
Total	3.5 (0.6)	(2.4-4.3)	(3.2-3.8)

**Figure 2:**
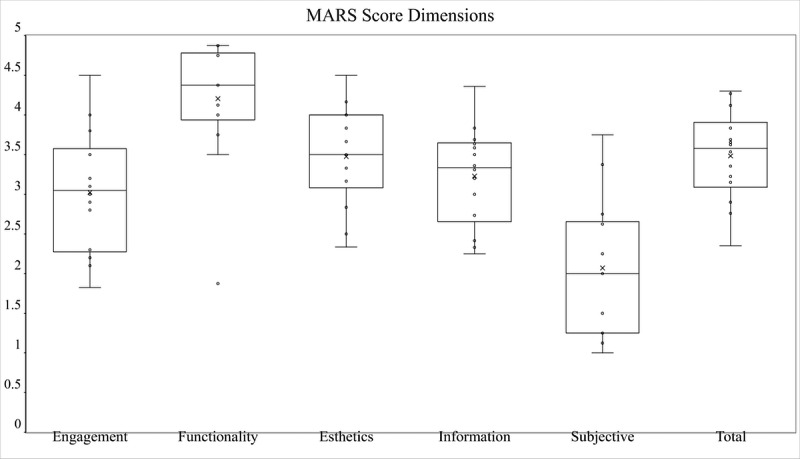
Mobile App Rating Scale (MARS) dimension scores.

The mean scores of the dimensions of the MARS are presented in Table 2. The values vary from “low” to “good.” The results of the one-way ANOVA reveal that there was at least one significant difference between the five dimensions regarding the score (*F*_5,78_=13.51, *P*<.001). In order to determine which mean values differed more specifically from each other, the Tukey test (HSD) was performed. The results showed that the functionality dimension value was significantly higher than the values of the other dimensions (*P*=.009). The results also showed that the subjective dimension value was significantly lower than the values of all other dimensions (*P*=.001). However, the difference in the mean values of the engagement, information, esthetic, and total dimensions was not significant (*P*=.09). The engagement dimension had an average score but had the most variability (median 3.1, IQR 1.3). Figure 2 shows box plots with the median, first and third quartiles, and minimum and maximum scores. Each point represents the score for each app.

### Therapeutic Quality: Behavior Change Techniques

The mean number of BCTs identified in the apps was 22 (SD 10), with a maximum of 38 techniques and a minimum of 4 techniques. The IRR between the two raters was almost perfect. The ICC was 0.92 (95% CI 0.89-0.93), prevalence and adjusted kappa was 0.85 (95% CI 0.84-0.85), and Krippendorf alpha was .85 (95% CI .79-.9). Detailed results are presented in Multimedia Appendix 3.

The significance in the Shapiro-Wilk test (*P*=.11) indicated the inability to assess the strengths of the different prevalences of BCTs observed in each app. Yet, delivery of the intervention (D), addressing motivation to stay a nonsmoker (M), specific behavior change techniques focused on smoking behavior (B), and maximizing self-regulatory capacity and skills (S) were observed most frequently (between 62% [8.7/14] and 64% [9/14] of the apps), whereas proposing adjuvant strategies (A) was least frequently observed (31% [4.3/14] of the apps) (Table 3) [27].

**Table 3:** Behavioral and cognitive techniques classified by function in the smoking cessation apps studied, according to the taxonomy of Michi et al [27].

BCTs^a^ classified by function	Mean (SD) app number (out of 14)		Range (minimum-maximum)	Percentage	
Delivery of the intervention (D) (ie, provide adapted behavioral support)	9.0 (1.1)	(1.5-13)		64%	
Addressing motivation to stay a nonsmoker (M) (ie, provide information of the advantages of quitting)	8.8 (3.5)	(1.5-12.5)		63%	
Specific smoking behavior change techniques (B) (ie, Monitor how the client achieves his goal)	8.7 (3.0)	(1.5-13)		62%	
Maximizing self-regulatory capacity and skills (S) (ie, provide advise on how to avoid social pressure)	8.6 (2.7)	(2.5-13)		62%	
Information gathering (I) (ie, evaluate the patient’s readiness to quit)	5.9 (2.2)	(3-7.5)		42%	
General communication (C) (ie, provide information on withdrawal symptoms)	5.8 (3.3)	(0-10)		41%	
General aspects of interaction (R) (ie, encourage or reassure on client experiences)	5.3 (3.1)	(0-10)		38%	
Adjuvant activities (A) (ie, explain the advantages of medication if needed)	4.3 (4.1)	(0-8.5)		31%	

^a^BCTs: behavioral and cognitive techniques.

### Relationship Between App Characteristics and Quality Level

Mean price per month, mean user rating, and number of BCTs were tested for correlation with the MARS score (Table 4). The mean MARS score (mean 3.5, SD 0.6) was positively correlated with price (mean 3.0, SD 3.4) (*r*=0.70, *P*=.007) and the number of implemented BCTs (mean 22, SD 11) (*r*=0.66, *P*=.01). User rating was not correlated with any quality scale (Table 4). The mean price per month (mean 3.0, SD 3.4) was positively correlated with the mean user rating (mean 4.5, SD 0.3) (*r*=0.58, *P*=.03) and the number of BCTs in the app (mean 22, SD 11) (*r*=0.60, *P*=.03).

**Table 4:** Correlations among the total Mobile App Rating Scale (MARS) score, price, user rating, and number of behavioral and cognitive techniques.

Variable		Mean price (€)	User mean rating score	MARS^a^ score	Total number of BCTs^b^
**Mean price (€)**					
	*r*	1	0.58	0.70	0.59
	*P* value	—^c^	.03	.007	.03
**User mean rating score**					
	*r*	0.58	1	0.098	0.124
	*P* value	.03	—	.74	.67
**MARS score**					
	*r*	0.70	0.098	1	0.66
	*P* value	.007	.74	—	.01
**Total number of BCTs**					
	*r*	0.59	0.124	0.66	1
	*P* value	.03	.67	.01	—

^a^MARS: Mobile App Rating Scale.

^b^BCTs: behavioral and cognitive technique.

^c^Not applicable.

## Discussion

### Systematic Search Results

The current review aimed to examine the content quality of popular mobile apps for smoking cessation in the French market. This type of content analysis is, to our knowledge, the first of its kind for the following two main reasons: the target market of the study and the methodology. French mHealth apps were reviewed in this study, with the aim to examine both of the following aspects of content quality: the general quality via the MARS scale and the therapeutic dimension though the behavior change technique taxonomy.

### General App Characteristics

Based on the established behavior change technique taxonomy and the MARS, we analyzed a total of 14 apps. It appears that the French mobile app market is less developed than the English one, where the number of reviewed apps is much higher. Indeed, 252 apps were identified in the US market in 2013 [11] and 225 apps were identified in 2016 [14]. Similar to these findings, 112 apps were examined in Australia [15] and 140 apps in England [16]. Consequently, even if several apps are available on the market, they are not accessible to most of the native French-speaking population speaking only French. This accessibility limitation could be overcome by translating existing apps on the market. However, the translation process would need to take into account the cultural context and be periodically adapted to each update to ensure users can access and benefit from the app content. Since this process would require human time and financial resources, the translation of apps could be decided based on supply and demand.

### General Quality: MARS

The general quality of popular apps in France varies from “acceptable” to “good.” In spite of these results, health professional judges do not recommend most of the apps. It seems the general quality threshold needed to be recommended is not met by most of the popular apps on the French market.

Our findings suggest that popular apps focused primarily on the functionality dimension that is composed of the following four aspects: performance, ease of use, navigation, and gestural design. The priority on the functionality dimension is a trend already observed for smoking cessation [29] and weight management [21] apps. Enhancing only this dimension will not be enough to improve the general quality of content. Nevertheless, we recognize the importance of the functionality dimension as a facilitator for the use of mHealth solutions. Better integration of clinical expertise seems to be necessary to create engaging and informative content.

### Therapeutic Quality: Behavior Change Techniques

The use of BCTs in our mobile app sample was not normally distributed, indicating that evidence-based techniques are not properly implemented in the French market. Our findings show that the market is still in the initial stages, most likely driven by technical expertise, and suggest that there is a lack of theory-driven behavioral change techniques, as proposed previously [16,29]. In France, mobile app development is not driven by BCT theory because its techniques are rarely invoked in popular apps. Indeed, even the most widely used BCT functions were absent in more than 65% of the sample.

The strength of current apps is their focus on the target behavior through addressing motivation and maximizing self-regulatory capacity and skills. In contrast, our results suggest that most apps failed to include features from the adjuvant activities dimension and focus on the general aspect of interaction. Failure to promote activities that indirectly facilitate abstinence (ie, inform or advise on medication to stop smoking) is consistent with the tendency in English-speaking countries [10,30]. A lack of adjuvant activities results in harsh deprivation considering that adherence to medications that help to stop smoking increases the likelihood of successful abstinence by 50% compared to “cold turkey” [2]. Additionally, our findings indicate a deficit of techniques necessary for effective delivery (ie, acquire and communicate relevant information needed to adapt the intervention). In addition, some authors pointed out the importance of technique interactions for an effective behavior change, promoting the idea that some BCTs can be effective solely under specific combinations (used at their best under specific conditions). As reported, the least effective interventions were those providing *feedback on performance* without *providing instructions* [31]. We therefore believe that the current strength of the French mHealth market can be severely undermined owing to the limited information and communication techniques used.

### Relationship Between App Characteristics and Quality Level

A noteworthy result was the positive relationship between both general and therapeutic qualities. This association is relevant since efficacy is influenced by not only the content of a therapy (in this case the therapeutic quality), but also adherence to treatment [18].

The second interesting result was the absence of correlation between the app store’s user rating and the qualities assessed, suggesting that user ratings may not be a good predictor of general or therapeutic quality. These results may give rise to concerns as users choose their apps based primarily on the ratings on the app store [32]. This highlights the importance of creating standards and accreditation for mHealth apps to protect users.

The third interesting result was the relation between price and both quality measurements (the cheaper the app, the poorer the qualities, with the exception of two apps financed by public institutions). This result is at odds with the supposed accessibility benefits of mobile apps [6], which casts a doubt on the benefits of affordability. To better understand this relationship, more studies that address the issue of intervention efficacy should address the issue of the cost-benefit ratio.

### Strengths and Limitations

An important strength of this study is that it is the first to examine smoking cessation apps on both the Apple Store and Google Play Store in the French market. The IRR was evaluated following the best practice recommendations for content analysis [28], and in all the tests, the IRR varied from substantial to almost perfect for each scale.

The findings of this study should be interpreted in light of some limitations. First, the apps were rated from the first and only use. There is a probability that some BCTs were not accessible to the raters and therefore were underrated. On the contrary, after prolonged use, apps could be seen as less engaging than in the first use, as the engagement attrition rate of mHealth apps is very high [33,34]. Second, we are aware of critics questioning the link between the quality and popularity of smoking cessation apps (whether the quality is therapeutic or general). Indeed, the few apps that exhibited high adherence to therapeutic guidelines were not necessarily the most popular [16], and about 17% of the high general quality apps identified appeared in the top 10 recommended smoking cessation apps in New Zealand app stores [15]. The aim of our study was not to identify the best solution in the French market, but to establish the quality of the most used apps available for French users.

### Conclusions

The general and therapeutic content quality of popular smoking cessation apps in France varied by app type and price. General and therapeutic contents are positively correlated. The user rating on app stores is not an indicator of the general and therapeutic content quality. The findings suggest that popular apps focused primarily on the functionality dimension. At least half of the apps addressed motivation and advised on using behavioral skills to quit smoking or stay a nonsmoker; however, only a handful of apps gathered important information and delivered proper advice regarding the use of approved medication or the implementation of behavioral techniques. Overall, the findings provide the first snapshot of the quality of popular smoking cessation apps in France. This review will need to be revised in order to examine whether the content quality of smoking cessation apps will evolve in the French market. Further research is needed to understand how users engage and benefit from these apps in the real world.

## Appendix

Multimedia Appendix 1 Detailed information of all apps included in the analysis.

Multimedia Appendix 2 Interrater reliability with 95% CI according to each dimension of the Mobile App Rating Scale (MARS).

Multimedia Appendix 3 Interrater reliability with 95% CI according to each dimension of the behavior change technique taxonomy.

## Abbreviations

ANOVA: analysis of variance
BCT: behavioral and cognitive technique
HSD: honestly significant difference
ICC: interclass correlation coefficient
IRR: interrater reliability
MARS: Mobile App Rating Scale
mHealth: mobile health
