# Supplementary material for: Behavior Change Techniques in Popular Mobile Apps for Smoking Cessation in France: Content Analysis
Source: JMIR Mhealth Uhealth. 2021 May 13;9(5):e26082. doi: 10.2196/26082 (PMC8160788; doi:10.2196/26082)
Supplement: Multimedia Appendix 1 [file mhealth_v9i5e26082_app1.docx]

**Multimedia Appendix 1.** Detailed information of all apps included in the analysis.

| Name app | App ID | MARS Score ^a^ | Price (€) ^b^ | BCTs ^c^ | User rating ^d^ |
| --- | --- | --- | --- | --- | --- |
| QuitNow | 1 | 3,7 | 4,2 | 14 | 4,5 |
| Smoke Free | 2 | 4,3 | 5,5 | 38 | 4,6 |
| Kwit | 3 | 4,1 | 10,0 | 30 | 4,5 |
| Stop-tabac | 4 | 2,3 | 0,0 | 20 | 4,3 |
| Tabac info service | 5 | 3,8 | 0,0 | 24 | 4,1 |
| Stop Smoking - EasyQuit free 7 | 6 | 3,4 | 5,2 | 21 | 4,8 |
| Ouiquit | 7 | 4,3 | 5,5 | 26 | 4,7 |
| Tabac.io | 8 | 2,9 | 0,0 | 4 | 4,6 |
| quit smoking slowly 29 | 9 | 3,2 | 0,0 | 17 | 4,2 |
| Quit Smoking Pro 32 | 10 | 3,2 | 0,0 | 5 | 4,2 |
| Flamy | 11 | 3,6 | 8,0 | 33 | 4,8 |
| Quitzilla | 12 | 2,8 | 0,0 | 14 | 4,7 |
| Quit Tracker Stop Smoking 0 | 13 | 3,7 | 3,0 | 31 | 4,7 |
| My Quit Smoking Coach 106 | 14 | 3,5 | 0,0 | 33 | 3,7 |

*Note.*

^a^ Mean score of MARS scale from 1 to 5

^b^ Average price per month in euros

^c^ Number of BCT techniques for smoking cessation out of 44

^d^ Average user rating on Google Store
