# Supplementary material for: Behavior Change Techniques in Popular Mobile Apps for Smoking Cessation in France: Content Analysis
Source: JMIR Mhealth Uhealth. 2021 May 13;9(5):e26082. doi: 10.2196/26082 (PMC8160788; doi:10.2196/26082)
Supplement: Multimedia Appendix 2 [file mhealth_v9i5e26082_app2.docx]

**Multimedia Appendix 2.** Interrater reliability with 95% CI according to each dimension of the Mobile App Rating Scale (MARS).

| MARS | Kalpha | ICC | Weighted Kappa |
| --- | --- | --- | --- |
| Engagement | 0,723 (0,554 - 0,858) | 0,843 (0,744 - 0,902) | 0,725 (0,594 - 0,856) |
| Functionality | 0,524 (0,327 - 0,696) | 0,756 (0,576 - 0,858) | 0,603 (0,408 - 0,799) |
| Aesthetics | 0,712 (0,516 - 0,805) | 0,839 (0,701 - 0,914) | 0,718 (0,574 - 0,863) |
| Information | 0,819 (0,742- 0,886) | 0,891 (0,837 - 0,937) | 0,823 (0746 - 0,900) |
| Subjective score | 0,691 (0,542 -0,804) | 0,821 (0,696 - 0,895) | 0,692 (0,540 - 0,845) |
| Total | 0,797 (0,748 - 0,841) | 0,885 (0,853 - 0,910) | 0,793 (0,741 - 0,846) |
