# Supplementary material for: Behavior Change Techniques in Popular Mobile Apps for Smoking Cessation in France: Content Analysis
Source: JMIR Mhealth Uhealth. 2021 May 13;9(5):e26082. doi: 10.2196/26082 (PMC8160788; doi:10.2196/26082)
Supplement: Multimedia Appendix 3 [file mhealth_v9i5e26082_app3.docx]

**Multimedia Appendix 3.** Interrater reliability with 95% CI according to each dimension of the behavior change technique taxonomy.

| **BCT** | **Kalpha** | **ICC** | **PABAK** |
| --- | --- | --- | --- |
| B | 0,806 (0,730 - 0,868) | 0,894 (0,862 - 0,918) | 0,806 (0,803 - 0,810) |
| M | 0,833 (0,736 - 0,916) | 0,910 (0,875 - 0,935) | 0,833 (0,828 - 0,838) |
| S | 0,780 (0,671 - 0,876) | 0,878 (0,824 - 0,915) | 0,780 (0,775 -0,785) |
| A | 0,90 (0,7667 - 1,00) | 0,948 (0,916 - 0,968) | 0,899 (0,892 - 0,907) |
| R | 0,826 (0,753 - 0,899) | 0,906 (0,868 - 0,932) | 0,826 (0,822 - 0,830) |
| I | 0,853 (0,707 - 0,963) | 0,922 (0,865 - 0,955) | 0,852 (0,844 - 0,861) |
| C | 0,818 (0,712 - 0,909) | 0,901 (0,854 - 0,932) | 0,818 (0,813 - 0,823) |
| D | 0,765 (0,452 - 1,0) | 0,879 (0,738 - 0,944) | 0,870 (0,719 - 0,940) |
| Total | 0,851 (0,796 - 0,9) | 0,920 (0,898 - 0,937) | 0,851 (0,848 - 0,854)  erreur (0,52) |
